# The association between gastric cancer and sarcopenia: a scoping review

**DOI:** 10.3389/fonc.2025.1684186

**Published:** 2025-10-16

**Authors:** Xue Wang, Xuefeng Sun, Yuanyu Wu, Yanjun Wang, Jingyi Ren, Xuedong Fang

**Affiliations:** ^1^ School of Nursing, Jilin University, Changchun, China; ^2^ School of Nursing, Changchun University of Chinese Medicine, Changchun, China; ^3^ China-Japan Union Hospital of Jilin University, Changchun, China; ^4^ Department of Gastrointestinal and Colorectal Surgery, The First Hospital of Jilin University, Changchun, China; ^5^ Nursing Department, The Bethune Hosptial Stomatology Jilin University, Changchun, China

**Keywords:** gastric cancer, stomach neoplasm, sarcopenia, sarcopenic obesity, prognosis

## Abstract

**Aim:**

To explore the relationship between gastric cancer and sarcopenia and review the underlying mechanisms.

**Method:**

A systematic search was conducted across the Web of Science, PubMed, Cochrane, CNKI, Wanfang, and VIP databases following the Preferred Reporting Items for Systematic Reviews and Meta-Analyses extension for Scoping Reviews (PRISMA-ScR) guidelines. Literature describing the relationship between gastric cancer and sarcopenia was included in this study, with methodological quality assessed using the Joanna Briggs Institute (JBI) Critical Appraisal Tools.

**Results:**

Among the 1,518 identified publications, 33 cohort studies involving 10,679 participants were ultimately included. The results revealed a sarcopenia prevalence ranging from 6.8% to 72.22% in gastric cancer patients. Most studies indicated that reduced muscle mass—potentially attributable to fat infiltration, immunosuppression, cachexia-associated metabolic disturbances, and protein reserve depletion—serves as an independent predictor of postoperative complications, overall survival, and disease-free survival in gastric cancer patients. However, due to heterogeneity in assessment criteria and measurement tools, only two studies demonstrated that sarcopenia did not significantly impact survival or prognosis in this population.

**Conclusion:**

Postoperative sarcopenia exhibits a high prevalence after gastric cancer surgery and is a significant predictor of adverse clinical outcomes. This underscores the importance of prioritizing muscle mass preservation in postoperative management and integrating its assessment into preoperative risk stratification. However, the current body of evidence is limited by inconsistent diagnostic criteria and a lack of mechanistic studies. Future research should focus on establishing standardized diagnostic frameworks through multidisciplinary collaboration and developing targeted interventions to improve patient prognosis.

## 1 Introduction

Gastric cancer, a malignancy originating from the gastric mucosal epithelium, represents a significant global health burden. Data indicate that an estimated 19.3 million new cancer cases occurred worldwide in 2020, with gastric cancer accounting for approximately 1.09 million cases (1). Cancer-related deaths approached 10 million, including roughly 769,000 gastric cancer fatalities, underscoring its persistent status as a major public health challenge globally (2). Patients with gastric cancer frequently experience persistent digestive dysfunction due to anatomical alterations, manifesting as chronic eating difficulties, vomiting, diarrhea, and malabsorption. Sarcopenia—a syndrome characterized by progressive loss of muscle mass and function—exhibits multifactorial pathogenesis involving chronic inflammation, malnutrition, mitochondrial dysfunction, prolonged disuse, neuromuscular degeneration, and insufficient physical activity (3).

Research demonstrates that tumor-associated inflammatory metabolic dysregulation and hypercatabolic states significantly contribute to sarcopenia pathogenesis in gastric cancer, with prevalence rates ranging from 10.0% to 57.7% (4). The underlying mechanisms involve proinflammatory cytokine-mediated enhancement of proteolytic pathways, where excessive IL-6 and TNF-α in the tumor microenvironment persistently activate both ubiquitin-proteasome and autophagy-lysosomal systems, accelerating muscle protein catabolism (5). Concurrently, tumor-induced insulin resistance and dysregulated lipid metabolism compromise bioenergetic supply to muscle tissue (6), while gastrointestinal obstruction and malabsorption further exacerbate protein-energy malnutrition, establishing a self-perpetuating vicious cycle (7). Notably, aberrant myokine secretion resulting from muscle atrophy modulates critical signaling pathways, including JAK/STAT and mTOR, thereby altering the tumor microenvironment to promote cancer proliferation and metastasis (8).

Despite accumulating evidence supporting the association between gastric cancer and sarcopenia, research on their bidirectional mechanisms remains fragmented due to methodological heterogeneity, population diversity, and lack of standardized interventions, precluding comprehensive systematic synthesis. This review, therefore, aims to consolidate existing evidence by integrating findings across study designs, analyzing how population characteristics modulate association strength, evaluating comparative merits of sarcopenia assessment tools, and elucidating the clinical implications of their interplay—ultimately informing the development of integrated management strategies encompassing screening, assessment, and targeted interventions for gastric cancer patients.

## 2 Materials and methods

This scoping review consolidates current knowledge on the sarcopenia-gastric cancer relationship through a five-phase methodology comprising research question development, systematic literature screening, rigorous study selection, standardized data extraction, and critical evidence synthesis (9), with all results reported in strict adherence to the Preferred Reporting Items for Systematic Reviews and Meta-Analyses Extension for Scoping Reviews (PRISMA-ScR) guidelines (10). The study protocol was registered on the Open Science Framework with the registration number https://doi.org/10.17605/OSF.IO/JC9VD.

### 2.1 Research questions

This review addresses three core research questions: 1. What is the reported prevalence range of sarcopenia among gastric cancer patients in existing studies? 2. How does sarcopenia affect survival and prognosis in gastric cancer patients? 3. What are the underlying biological mechanisms governing the bidirectional relationship between gastric cancer and sarcopenia?

### 2.2 Search strategy

This study conducted a systematic literature search under the guidance of a professional librarian, encompassing records from database inception to 1 July 2025, across PubMed, Web of Science, Embase, Cochrane Library, CNKI, Wanfang, and VIP databases, utilizing the key terms “gastric cancer” and “sarcopenia” as primary search parameters (**Table 1**).

**Table 1:** Medical subject headings (MeSH) and keywords used in searches.

#	MeSH	Free-text terms (in titles/abstracts)
#1	(“Stomach Neoplasm”) OR	(“Gastric Neoplasms” OR “Gastric Neoplasm” OR “Neoplasm, Gastric” OR “Neoplasms, Gastric” OR “Neoplasms, Stomach” OR” Cancer of Stomach OR “Stomach Cancers” OR “Cancer of the Stomach” OR Gastric Cancer” OR “Cancer, Gastric” OR “Cancers, Gastric” OR “Gastric Cancers” OR “Stomach Cancer” OR “Cancers, Stomach” OR “Cancer, Stomach” OR “Gastric Cancer, Familial Diffuse”)
#2	(“Sarcopenia”) OR	(“Muscular Atrophies” OR “Muscle Atrophies” OR “Muscle Atrophy” OR “Neurogenic Muscular Atrophy” OR “Neurogenic Muscular Atrophies” OR “Neurotrophic Muscular Atrophy” OR “Muscular Atrophy”)
#3		#1 AND #2

### 2.3 Study selection

Literature management and screening were performed using Zotero software. Inclusion criteria comprised (1) POS framework adherence: P (Participants)—adults (≥18 years) with clinically confirmed gastric cancer and sarcopenia; O (Outcomes)—gastric cancer-related complications, survival outcomes, and prognosis; S (Study design)—empirical human studies (randomized controlled trials, cohort studies, case-control studies, cross-sectional studies); (2) no restrictions on demographic characteristics or geographical regions. Exclusion criteria included (1) non-empirical studies (e.g., reviews, editorials, theoretical articles), (2) secondary data analyses, (3) non-English literature, and (4) studies failing to report outcomes examining the gastric cancer-sarcopenia relationship.

### 2.4 Data extraction

This study implemented a standardized data extraction protocol whereby two researchers independently extracted literature information using predefined Excel templates, with discrepancies resolved by a third reviewer. Extracted variables included first author, publication year, study design, country, gastric cancer staging, sample size, patient age, sarcopenia diagnostic criteria, assessment metrics, gastric cancer patient outcomes, and their interrelationship.

### 2.5 Evidence synthesis

Data were categorized according to research context, sample characteristics, assessment tools, metrics, outcome presentations, and key findings, with this review specifically centering on elucidating the bidirectional relationship between gastric cancer and sarcopenia.

### 2.6 Critical appraisal of included studies

The methodological quality of included studies was assessed using the Joanna Briggs Institute (JBI) Critical Appraisal Tools (11). As all incorporated studies were cohort designs, the corresponding checklist containing 11 appraisal items was applied.

## 3 Results

### 3.1 Search results and literature characteristics

**Figure 1:**
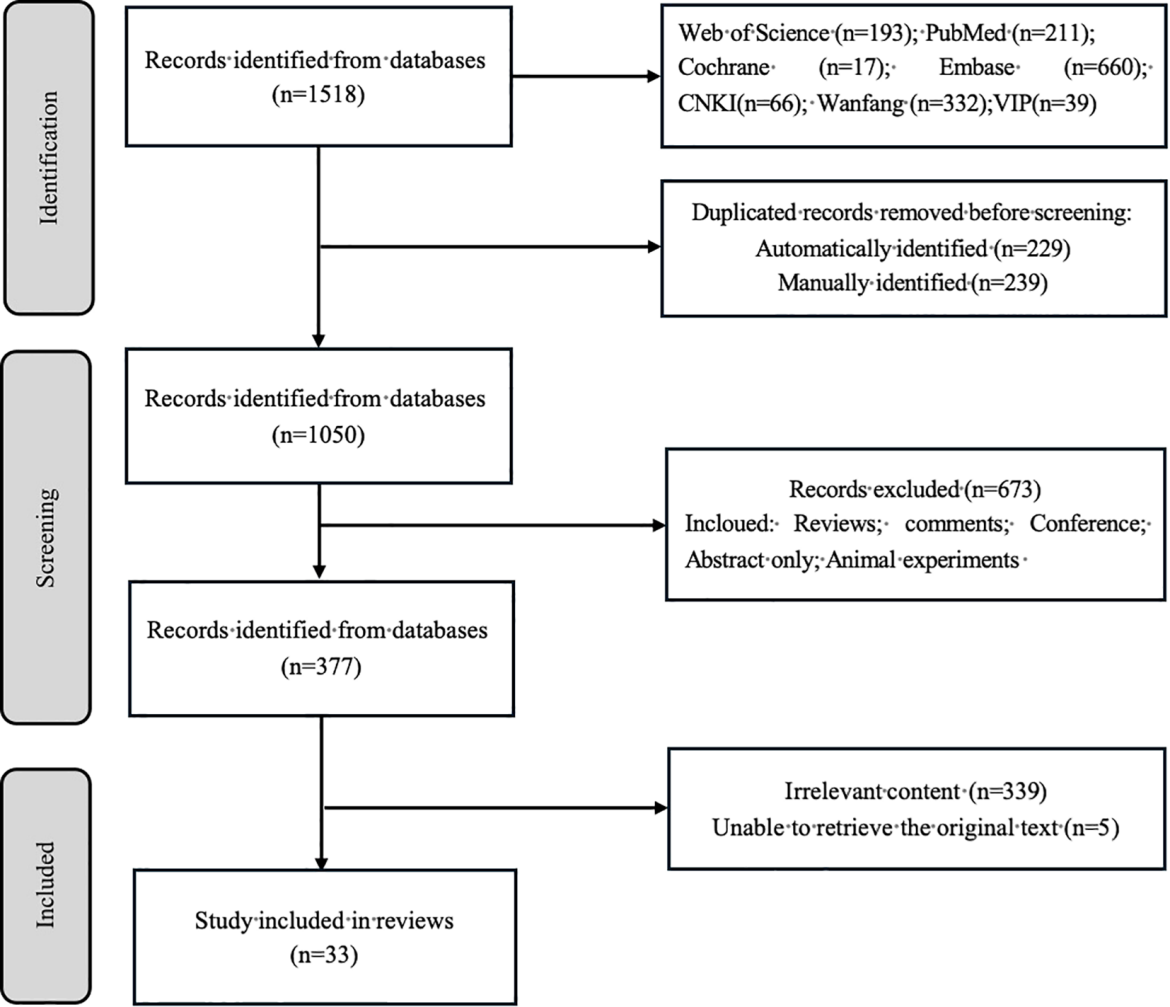
PRISMA-ScR flow diagram.

A comprehensive search identified 1,518 publications. Following deduplication (*n* = 468 excluded), title/abstract screening eliminated 673 records, yielding 377 articles for full-text assessment. Ultimately, 33 studies met the inclusion criteria and were incorporated into this review. The selection process is detailed in **Figure 1**. Quality appraisal confirmed that all eligible studies were retained for analysis (Appendix 1).

Geographically, 25 studies originated from Asian countries, including 13 from China (14, 18–21, 23, 24, 27, 34, 35, 38, 42, 43), 8 from Japan (12, 16, 25, 29–33), 2 from South Korea (26, 36), and 2 from India (39, 41). European contributions comprised eight studies: Spain (*n* = 2) (15, 44), Italy (*n* = 2) (22, 38), with single studies from Turkey (13), Poland (17), Ireland (37), and Romania (40). Publications spanned 2016–2025, encompassing 10,679 participants aged 26–89 years. All studies employed cohort designs, with 23 retrospective cohorts (12, 14–18, 21, 22, 24–26, 28–37, 40, 44) and 10 prospective cohorts (13, 19, 20, 23, 27, 38, 39, 41–43). Regarding therapeutic approaches, surgery was reported as the primary gastric cancer treatment in most studies, while only three investigations incorporated chemotherapy (28, 31, 36)—including one combining surgical and chemotherapeutic approaches (31) (**Tables 2**, **3**).

**Table 2:** Characteristics and analytics of included studies (*N* = 33).

Author(s) year, country	Study design	Sample size/age	Treatment method	Sarcopenia				
				Assessment indicators	Diagnostic criteria	Measure	Threshold definition	Prevalence
Uchida et al., 2021 (Japan) (12)	Retrospective cohort study	*N* = 353 64–78	Surgery	SMI, IMAC	NA	① CT ②SYNAPSE and Volume Analyzer	**SMI (cm²/m²)** Male: < 41.6; Female: < 34.1	NA
Erkul et al.,2022 (Turkey) (13)	Prospective cohort study	*N* = 146 63.8 ± 11.6	Surgery	Muscle mass Muscle strength Physical performance	EWGSOP	① CT ② Handgrip strength ③ 4 m usual gait speed	**Muscle mass (cm²/m²)** Male: BMI < 25, SMI < 43; BMI > 25, SMI < 53; Female: SMI < 41 **Muscle strength** Male: < 27 kg; Female: < 16 kg **Physical performance:** gait speed < 0.8 m/s	21.2%
Ma et al., 2019 (China) (14)	Retrospective cohort study	*N* = 545 62.62 ± 10.53	Surgery	SMI Muscle strength Physical performance	EWGSOP	① CT ② Handgrip strength ③ 6 m usual gait speed	**Muscle mass (cm²/m²)** Male: SMI < 40.8; Female: SMI < 34.8 **Muscle strength** Male: < 26 kg; Female: < 18 kg **Physical performance:** gait speed < 0.8 m/s	7.3%
Juez et al.,2023 (Spain) (15)	Retrospective cohort study	*N* = 190 72 ± 11.1	Surgery	SMI VAT	CT	CT	**SMI (cm²/m²)** Male: BMI < 25, SMI < 43; BMI > 25, SMI < 53; Female: SMI < 41 VAT: -150 ~ -50HU	Sarcopenia (14.7%) SO (21.1%)
Sugawara et al., 2020 (Japan) (16)	Retrospective cohort study	*N* = 1166 NA	Surgery	SMI	CT	CT	**SMI (cm²/m²)** Male: SMI < 43.78; Female: SMI < 35.30	23.8%
Sierzega et al., 2019 (Poland) (17)	Retrospective cohort study	*N* = 138 63 (26–87)	Surgery	SMI	International consensus definitions	CT	**SMI (cm²/m²)** Male: SMI < 52.4; Female: SMI < 38.5	43%
Zheng et al., 2024 (China) (18)	Retrospective cohort study	*N* = 781 61.1 ± 11.3	Surgery	SMI	CT	CT	**SMI (cm²/m²)** Male: SMI < 36.4; Female: SMI < 28.4	26.5%
Wang et al., 2016 (China) (19)	Prospective cohort study	*N* = 255 65.14 ± 10.81	Surgery	SMI Muscle strength Physical performance	EWGSOP AWGS	① CT ② Handgrip strength ③ 6 m usual gait speed	**SMI (cm²/m²)** Male: SMI < 36.0; Female: SMI < 29.0 **Muscle strength** Male: < 26 kg; Female: < 18 kg **Physical performance:** gait speed ≤ 0.8 m/s	12.5%
Lou et al., 2016 (China) (20)	Prospective cohort study	*N* = 206 64.05 ± 10.1	Surgery	SMI Muscle strength Physical performance	EWGSOP AWGS	① CT ② Handgrip strength ③ 6 m usual gait speed	**SMI (cm²/m²)** Male: SMI < 40.8; Female: SMI < 34.9 **Muscle strength** Male: < 26 kg; Female: < 18 kg **Physical performance:** gait speed < 0.8 m/s	6.8%
Duan et al., 2024 (China) (21)	Retrospective cohort study	*N* = 207 59.6 ± 10.2	NAC Surgery	SMI VFA	CT	CT	**SMI (cm²/m²)** Male: SMI < 52.4; Female: SMI < 38.5 VFA: -150 ~ -50HU	Sarcopenia (37.7%) SO (25.1%)
Ricciardolo et al., 2022 (Italy) (22)	Retrospective cohort study	*N* = 55 69.89 ± 11.1	Surgery	SMI	CT	CT	**SMI (cm²/m²)** Male: SMI < 52.4; Female: SMI < 38.5	70%
Zhang et al., 2022 (China) (23)	Prospective cohort study	*N* = 507 63	Surgery	SMI Muscle strength SMD	EWGSOP	CT	**SMI (cm²/m²)** Male: SMI < 40.8; Female: SMI < 34.9 **Muscle strength** Male: < 26 kg; Female: < 18 kg **SMD (HU):** Male: < 38.5; Female: < 28.6	14.4%
Ding et al., 2024 (China) (24)	Retrospective cohort study	*N* = 381 58.5	Robotic surgery	SMI	CT	CT	**SMI (cm²/m²)** Male: SMI < 40.8; Female: SMI < 34.9	70.4%
Tamura et al., 2019 (Japan) (25)	Retrospective cohort study	*N* = 153 Sarcopenic: 74 Nonsarcopenic: 68	Surgery	Body composition MMI	NA	Multifrequency BIA	**MMI (cm²/m²)** Male: < 15.44; Female: < 13.33	15.7%
Kim et al., 2020 (Korea) (26)	Retrospective cohort study	*N* = 305 58.7 ± 11.9	Surgery	SMI BMI	CT WHO	CT	**SMI (cm²/m²)** Male: SMI < 56.2; Female: SMI < 53.6 **BMI (kg/m²)** underweight: BMI< 18.5; normal: 18.5< BMI < 23; overweight: BMI ≥ 23	37.7%
Zhang et al., 2018 (China) (27)	Prospective cohort study	*N* = 156 59.1 ± 9.9	Surgery	SMI	CT	CT	**SMI (cm²/m²)** Male: SMI < 40.8; Female: SMI < 34.9	15.4%
Zurlo et al., 2024 (Italy) (28)	Retrospective cohort study	*N* = 88 57 (30–78)	Chemotherapy	SMI	CT	CT	**SMI (cm²/m²)** Male: SMI < 55; Female: SMI < 39	60.2%
Kouzu et al., 2021 (Japan) (29)	Retrospective cohort study	*N* = 67 70.8 ± 8.3	Surgery	PMI	CT	CT	**PMI (cm²/m²)** Male: PMI < 0.766; Female: PMI < 0.704	37.3%
Matsui et al., 2021 (Japan) (30)	Retrospective cohort study	*N* = 840 Low-IMAC: 63.09 ± 11.8 High-IMAC: 69.91 ± 9.24	Surgery	IMAC SMI VFA	CT	CT	**IMAC (cm²/m²)** Male: -0.430; Female: -0.310 **SMI (cm²/m²)** Male: 43.08; Female: 33.73 VFA: -150 ~ -50HU	50.2%
Matsunaga et al., 2021 (Japan) (31)	Retrospective cohort study	*N* = 67 67.6 ± 9.9	Surgery Chemotherapy	SMI	CT	CT	**SMI (cm²/m²)** Male: 43.9; Female: 34.7	49.3%
Tanaka et al., 2023 (Japan) (32)	Retrospective cohort study	*N* = 150 69 (34–88)	Surgery	SMI MR	CT	CT	**SMI (cm²/m²):** Male: 36.4; Female: 31.2 **MR: 14%**	23.3%
Dogan et al., 2024 (Japan) (33)	Retrospective cohort study	*N* = 118 63 (27–89)	Surgery	HUAC	CT	CT	Male: 10.45HU; Female: 9HU	24.6%
Zhong et al., 2024 (China) (34)	Retrospective cohort study	*N* = 717 62 (55–67)	Surgery	SMI SMRA SMG	CT	CT	**SMI (cm²/m²):** 45 **SMRA:** 45HU **SMG:** SMI × SMRA = 2025	NA
Li et al., 2025 (China) (35)	Retrospective cohort study	*N* = 198 58.9	Surgery	SMI SML	EWGSOP	CT	**SMI (cm²/m²)** Male: SMI < 40.8; Female: SMI < 34.9	Preoperative: 23.7% Postoperative: 33.3%
Lee et al., 2018 (Korea) (36)	Retrospective cohort study	*N* = 140 Sarcopenic: 69 Nonsarcopenic: 66	Palliative chemotherapy	SMI	KNHANES	CT	**SMI (cm²/m²)** Male: SMI < 49; Female: SMI < 31	47.9%
O’Brien et al., 2018 (Ireland) (37)	Retrospective cohort study	*N* = 56 68.4 ± 11.9	Surgery	SMI	CT	CT	**SMI (cm²/m²)** Male: SMI < 52.4; Female: SMI < 38.5	35.7%
Wu et al., 2025 (China) (38)	Prospective cohort study	*N* = 1654 66 (14)	Surgery	SMI Muscle strength Physical performance Muscle-specific strength	GLIS EWGSOP AWGS	① CT ② Handgrip strength ③ 6 m usual gait speed	**SMI (cm²/m²)** Male: SMI < 40.8; Female: SMI < 34.9 **Muscle strength** Male: < 28 kg; Female: < 18 kg **Physical performance** gait speed < 1 m/s	Criteria 1: 24.2% Criteria 2: 17.0% Criteria 3: 32.5%
Wagh et al., 2024 (India) (39)	Prospective cohort study	*N* = 68 55.86	Surgery	SMI Muscle strength	AWGS	① CT ② Handgrip strength	**SMI (cm²/m²)** Male: SMI < 40.8; Female: SMI < 34.9 **Muscle strength** Male: < 28 kg; Female: < 18 kg	42.3%
Beuran et al., 2018 (Romania) (40)	Retrospective cohort study	*N* = 78 67.7 ± 12.7	Surgery	SMI	CT	CT	**SMI (cm²/m²)** Male: SMI < 52.4; Female: SMI < 38.5	72.22%
Bhattacharyya et al., 2022 (India) (41)	Prospective cohort study	*N* = 72 55.67 ± 11.15	Surgery	SMI Muscle strength	CT	①CT ②Handheld dynamometer	**SMI (cm²/m²)** Male: SMI < 52.4; Female: SMI < 38.5 **Muscle strength** Male: < 30 kg; Female: < 20 kg	50%
Chen et al., 2024 (China) (42)	Prospective cohort study	*N* = 289 67.6 ± 11.4	Surgery	SMI VFA	CT	CT	**SMI (cm²/m²)** Male: SMI < 40.02; Female: SMI < 32.05 **VFA (cm²)** Male: SMI < 126.3; Female: SMI < 72.42	7.61%
Zhao et al., 2025 (China) (43)	Prospective cohort study	*N* = 335 67 (15)	Surgery	SMI SMD	EWGSOP	CT	**SMI (cm²/m²)** Male: SMI < 40.8; Female: SMI < 34.9 **SMD (HU):** Male: < 38.5; Female: < 28.6	NA
Rodrigues et al., 2021 (Spain) (44)	Retrospective cohort study	*N* = 198 73.5	Surgery	SMI VFA	CT	CT	**SMI (cm²/m²)** Male: SMI < 52.4; Female: SMI < 38.5 **VFA (cm²)** Male: SMI > 163.8; Female: SMI < 80.1	Sarcopenia (45.4%) SO (28%)

GC, gastric cancer; SMI, Skeletal Muscle Index; IMAC, intramuscular adipose tissue content; EWGSOP, European Working Group on Sarcopenia in Older People; VAT, visceral adipose tissue; HU, Hounsfield; SO, Sarcopenic Obesity; AWCS, Asian Working Group for Sarcopenia; NAC, neoadjuvant chemotherapy; OS, overall survival; RFS: relapse-free survival; VFA, visceral fat area; SMD, muscle quality; DFS, disease-free survival; MMI, muscle mass index; RBP, retinol-binding protein; PFS, progression-free survival; PMI, psoas muscle index; CKD, chronic kidney disease; MR, muscle reduction; (MR) was calculated as follows: (preoperative SMI - SMI 1 year after surgery)/preoperative SMI×100%; HUAC, calculation of the average Hounsfield units; SMRA, skeletal muscle radiation attenuation; SMG, skeletal muscle gauge; AWGS, Asian Working Group for Sarcopenia; AML, skeletal muscle loss [(SMI^post−op^– SMI^pre−op^)/SMI^pre−op^)× 100]; KNHANES, Korea National Health and Nutrition Examination Study; GLIS, Global Leadership Initiative in Sarcopenia; Criteria 1, low muscle-specific strength; Criteria 2, low muscle strength plus low muscle mass; Criteria 3, low muscle strength plus either low muscle mass or low muscle-specific strength.

**Table 3:** The relationship between GC and sarcopenia (*N* = 33).

Author(s) year, country	Sample size/age	Treatment method	GC		Relationship between GC and sarcopenia	Key findings
			Outcome assessment/prevalence	Follow-up		
Uchida et al., 2021 (Japan) (12)	*N* = 353 64–78	Surgery	Postoperative complication (infectious). 16.7%	30 days	High IMAC (OR = 2.391, *P* = 0.011) Pneumonia: (*P* = 0.003) Anastomotic leakage: (*P* = 0.003)	A high IMAC was significantly correlated with infectious complications following gastrectomy for gastric cancer.
Erkul et al.,2022 (Turkey) (13)	*N* = 146 63.8 ± 11.6	Surgery	Postoperative complication Sarcopenic group: 54.8% Nonsarcopenic group: 25.2%	30 days	Postoperative overall complication was significantly higher in sarcopenic group than nonsarcopenic group (*P* = 0.003); Sarcopenia was identified as an independent risk factor for complications (OR = 2.73, *P* = 0.047)	Severe sarcopenia may serve as a more robust prognostic indicator. The variation between the complication rates for sarcopenic versus nonsarcopenic patients was mainly due to the difference in systemic complications.
Ma et al., 2019 (China) (14)	*N* = 545 62.62 ± 10.53	Surgery	Postoperative complication 27.5%	30 days	Sarcopenia was an independent predictor (OR = 2.330; 95%CI: 1.132 to 4.796; *P* = 0.022) for postoperative complications.	Sarcopenia is a significant independent risk factor for postoperative complications after gastrectomy in patients without nutritional risk.
Juez et al.,2023 (Spain) (15)	*N* = 190 72 ± 11.1	Surgery	Postoperative complication 42%	34.3 months	SO was identified as a risk factor for serious complications [OR = 2.82 (1.1–7.1); *P* = 0.028]; SO and VAT showed marginal significance in postoperative mortality (*P* = 0.056).	SO was a risk factor for severe postoperative complications as well as worse long-term oncological after a gastrectomy for GC. Early detection and treatment could improve GC outcomes.
Sugawara et al., 2020 (Japan) (16)	*N* = 1166 NA	Surgery	OS (5-year) Sarcopenic group: 71.3% Nonsarcopenic group: 89.0%	82.2 months	Sarcopenic patients had significantly worse 5-year overall survival than nonsarcopenic patients (*P* < 0.001).	Preoperative low nutritional status, especially when present in combination with sarcopenia, is associated with poor survival outcomes in patients with GC.
Sierzega et al., 2019 (Poland) (17)	*N* = 138 63 (26–87)	Surgery	**Sarcopenic group** Postoperative complication (43%) OS (11 months); Reoperations (23%) Hospital stay (8 days) **Nonsarcopenic group** Postoperative complication (23%) OS (36.7 months); Reoperations (9%) Hospital stay (6.5 days)	30 months	Postoperative complication (*P* = 0.011); Reoperations (*P* = 0.020); Hospital stay (*P* = 0.010); OS (*P* = 0.005). Sarcopenia remained an independent prognostic factor with an odds ratio of 1.94 (95% CI: 1.08 to 3.48; *P* = 0.026).	SMI, is associated with an increased risk of postoperative morbidity and impaired long‐term survival.
Zheng et al., 2024 (China) (18)	*N* = 781 61.1 ± 11.3	Surgery	**Sarcopenic group** OS (39.61%); RFS (39.61%) **Nonsarcopenic group** OS (58.71%); RFS (57.84%)	10 years	OS: (HR = 1.467, 95% CI: 1.169–1.839) RFS: (HR = 1.450, 95% CI: 1.157–1.819) In the 1st postoperative year, the risk of death (HR = 2.62; 95% CI: 1.581–4.332) and recurrence (HR = 2.34; 95% CI: 1.516–3.606) was the highest.	Sarcopenia remained an independent risk factor for postoperative very long-term prognosis of GC. The effect of sarcopenia on the long-term outcome of patients with GC was consistent at 10 years postoperatively.
Wang et al., 2016 (China) (19)	*N* = 255 65.14 ± 10.81	Surgery	**Sarcopenic group** Major postoperative complication (43.8%); hospital stay (16 days) **Nonsarcopenic group** Major postoperative complication (14.3%); hospital stay (13 days)	30 days	Sarcopenia was independent predictors of postoperative complications (*P* < 0.001),	Sarcopenic patients had adverse clinical outcomes and sarcopenia was an independent predictor of postoperative complications after gastrectomy.
Lou et al., 2016 (China) (20)	*N* = 206 64.05 ± 10.1	Surgery	**Sarcopenic group** Postoperative complication (64.29%) Hospital stay (17 days); Costs (68,026¥) **Nonsarcopenic group** Postoperative complication (23.96%) Hospital stay (13 days); Costs (55,316¥)	30 days	The incidence of postoperative complications was significantly higher in the sarcopenic group compared to the nonsarcopenic group (*P* = 0.003). Patients with sarcopenia incurred higher hospitalization costs (*P* = 0.003).	Although the prevalence of sarcopenia in overweight/obese patients is relatively low, its presence increases the risk of postoperative complications by sixfold.
Duan et al., 2024 (China) (21)	*N* = 207 59.6 ± 10.2	NAC Surgery	**Pre-NAC sarcopenic obesity** 30-day complication rate (36.4%) OS (46.2%); RFS (39.3%) **Pre-NAC nonsarcopenic obesity** OS (61.3%); RFS (55.4%)	52 months	The 3-year OS (*P* = 0.027) and RFS (*P* = 0.015) rates were significantly lower in patients who underwent pre-NAC SO than in those who did not.	SO was independently associated with both postoperative complications and survival outcomes, with significantly differential impacts on short-term and long-term outcomes.
Ricciardolo et al., 2022 (Italy) (22)	*N* = 55 69.89 ± 11.1	Surgery	**Sarcopenic group** OS (30.15 ± 24.9 months); RFS (24.62 ± 24.4 months) **Nonsarcopenic group** OS (48.94 ± 31.15 months); RFS (48.31 ± 31.9 months)	10 years	A statistically significant difference between the sarcopenic and nonsarcopenic groups was observed in terms of mean OS (*P* < 0.026) and mean RFS (*P* < 0.023).	Sarcopenia can be considered a critical risk factor for survival in patients with resectable GC treated with up-front surgery.
Zhang et al., 2022(China) (23)	*N* = 507 63	Surgery	**Sarcopenic group** Postoperative complication (32.9%) Hospital stay (13 days); Costs (65,210¥) One-year mortality (8.2%) **Nonsarcopenic group** Postoperative complication (17.5%) Hospital stay (12 days); Costs (55,197¥) One-year mortality (1.8%)	38.8 months	Patients with sarcopenia had significantly higher incidence of postoperative complications (*P* = 0.002), longer postoperative hospital stays (*P* < 0.001), higher hospitalization costs (*P* < 0.001) and one-year mortality (*P* = 0.002). Sarcopenia was the only independent risk factor of postoperative complications.	Sarcopenia was an independent risk factor for both short- and long-term clinical outcomes. Low muscle quantity and low handgrip strength mediated the adverse impacts of sarcopenia on postoperative complications while low muscle quality mediated the adverse impacts of sarcopenia on OS.
Ding et al., 2024 (China) (24)	*N* = 381 58.5	Robotic surgery	**Sarcopenic group** Overall complication (53.7%) OS: (85.0%); DFS (80.3%) **Nonsarcopenic group** Overall complication (21.1%) OS: (93.2%); DFS (88.4%)	23.4 months	Sarcopenia was a major independent risk factor for postoperative complications (OR = 3.66, 95% CI: 2.18–6.13, *P* < 0.001), OS (HR = 2.53, 95% CI: 1.19–5.40, P = 0.016), and DFS (HR = 1.99, 95% CI: 1.09–3.66, *P* = 0.026).	Preoperative sarcopenia is correlated with increased postoperative complications and poorer long-term survival in GC patients.
Tamura et al., 2019 (Japan) (25)	*N* = 153 Sarcopenic:74 Nonsarcopenic: 68	Surgery	**Sarcopenic group** Postoperative complication (37.5%) Infections complication (29.2%) **Nonsarcopenic group** Postoperative complication (16.3%) Infections complication (10%)	30 days	Postoperative complications occurred significantly more frequently in the group with sarcopenia (*P* = 0.024). Infectious complications occurred significantly more frequently in the sarcopenia group (*P* = 0.021). Sarcopenia was (OR = 3.674, *P* = 0.021) associated with postoperative infectious complication.	Sarcopenia is an independent risk factor for postoperative infectious complications in GC patients and can serve as a predictive indicator for postoperative infectious complications following GC surgery.
Kim et al., 2020 (Korea) (26)	*N* = 305 58.7 ± 11.9	Surgery	The 5-year OS was 81%. OS in the high SMI group showed a trend toward being higher than that in the low SMI group (*P* = 0.058)	59.5 months	High and low BMI, and low SMI, were independent prognostic factors for OS (HR = 2.355, 1.736, and 1.607, respectively; *P* = 0.009, 0.023, and 0.033, respectively).	SMI and BMI did not impact perioperative morbidity in patients undergoing gastrectomy for GC. Both SMI and BMI are useful prognostic factors for OS in GC.
Zhang et al., 2018 (China) (27)	*N* = 156 59.1 ± 9.9	Surgery	**Sarcopenic group** Overall complication (62.5%) **Nonsarcopenic group** Overall complication (27.3%)	NA	Sarcopenia was independently associated with overall complications (OR: 3.4; 95% CI: 1.3 to 8.8; *P* = 0.013). In patients with sarcopenia, serum RBP reached a lower level and recovered later (*P* = 0.007).	Sarcopenia is significantly associated with increased postoperative complication rates following radical gastrectomy for GC and adversely affects patients’ postoperative nutritional and inflammatory status.
Zurlo et al., 2024 (Italy) (28)	*N* = 88 57 (30–78)	Chemotherapy	**Sarcopenic group** PFS: (3.5 months); OS: (15 months) **Nonsarcopenic group** PFS: (6 months); OS: (20 months)	42 months	PFS was significantly higher in the nonsarcopenic population than in the sarcopenic group (HR = 0.52; 95% CI: 0.20–0.96; *P* = 0.04). There was no statistically significant difference in OS between the two groups (HR = 0.82; 95% CI: 0.41–1.63; *P* = 0.55).	Early recognition of sarcopenia may contribute to personalizing second or further lines of treatment in advanced GC.
Kouzu et al., 2021 (Japan) (29)	*N* = 67 70.8 ± 8.3	Surgery	**Sarcopenic group** OS: (118, 43.5–180.5 days) Survival rate: 3−year OS 6.0% **Nonsarcopenic group** OS: (300, 133.8–636.3 days) Survival rate: 3−year OS 21.0%	NA	The sarcopenia group had a significantly shorter OS from recurrence than did the nonsarcopenia group (*P* < 0.001). The survival rate from the time of recurrence in the sarcopenia group was significantly worse than that in the nonsarcopenia group (*P* < 0.001). Sarcopenia was independent unfavorable prognostic factors (HR = 5.04).	Sarcopenia was poor prognostic factors after GC recurrence. To improve prognosis, preventing sarcopenia development after gastrectomy is required.
Matsui et al., 2021 (Japan) (30)	*N* = 840 High-IMAC group 69.91 ± 9.24 Low-IMAC group 63.09 ± 11.8	Surgery	**High-IMAC** Total complications [91 (21.6%)] Infectious complications [73 (17.3%)] **Low-IMAC** Total complications [63 (15.1%)] Infectious complications [49 (11.7%)]	NA	High-IMAC was an independent risk factor for severe complications (OR: 2.260, 95% CI: 1.220–4.190, *P* = 0.010). High IMAC showed significant associations with intra-abdominal infections (*P* = 0.052) and anastomotic leakage (*P* = 0.034)	Muscle quality (regardless of the assessment method used) is a significant predictor of postoperative complications.
Matsunaga et al., 2021 (Japan) (31)	*N* = 67 67.6 ± 9.9	Surgery Chemotherapy	**SMI^Low^ group** Survival rate (15.8 months) Side effects (63.6%) **SMI^High^ group** Survival rate (17.8 months) Side effects (32.4%)	NA	The SMI^Low^ group had a significantly higher incidence of grade 3 or 4 side effects (*P* = 0.010). The median survival rate was significantly higher in the SMI^High^ group (*P* = 0.034). SMI was an independent prognostic factor (*P* = 0.037).	The incidence of grade 3 or 4 side effects was significantly higher in patients with SMI^Low^ recurrent GC. SMI was a useful prognostic marker of recurrent GC.
Tanaka et al., 2023 (Japan) (32)	*N* = 150 69 (34–88)	Surgery	**Low SMIg/High MR group** OS (20%) **High SMI/Low MR group** OS (88.8%)	57 months	SMI (HR = 0.927; 95% CI = 0.877–0.979, *P* = 0.0066) and MR (HR = 0.954; 95% CI = 0.917–0.992, *P* = 0.0177) were independent prognostic factors.	Reduction in skeletal muscle mass after GC surgery were significantly associated with overall survival. Long-term management of these patients should focus on maintenance of postoperative skeletal muscle mass.
Dogan et al., 2024 (Japan) (33)	*N* = 118 63 (27–89)	Surgery	**Sarcopenic group** OS: (2 months) **Nonsarcopenic group** OS: (10 months)	43 months	Patients with sarcopenia demonstrated significantly shorter median survival compared to non-sarcopenic patients (*P* < 0.001).	Sarcopenia may impact the survival prognosis of patients with metastatic GC.
Zhong et al., 2024 (China) (34)	*N* = 717 62 (55–67)	Surgery	**Low SMG group** Postoperative complication (27.6%) 3-year OS: (51%); 3-year DFS (41%) 3-year RFS (41%) **High SMG group** Postoperative complication (9.3%) 3-year OS: (89%); 3-year DFS (86%) 3-year RFS (85%)	36 months	SMG is an independent protective factor against postoperative complications (OR = 0.98, 95%CI:0.97–0.99). SMG was better than SMI and SMRA in predicting OS, DFS, and RFS among the three groups of muscle parameters [SMI, 0.743; SMRA, 0.610; SMG, 0.761], DFS [SMI, 0.720; SMRA, 0.598; SMG, 0.728], RFS [SMI, 0.718; SMRA, 0.622; SMG, 0.755]	SMG is a powerful indicator for predicting the prognosis of GC patients. Its efficacy in predicting postoperative complications and long-term survival is significantly superior to that of a single muscle quantity indicator (SMI) or quality indicator (SMRA). As a comprehensive muscle parameter, SMG has higher clinical predictive value.
Li et al., 2025 (China) (35)	*N* = 198 58.9	Surgery	**Preoperative sarcopenic group** Total complications (31.91%) 5-year OS (38.3%) **Preoperative nonsarcopenic group** Total complications (13.25%) 5-year OS (56.95%) **Postoperative sarcopenic group** 5-year OS (34.25%) **Postoperative nonsarcopenic group** 5-year OS (63.2%)	68.9 months	Preoperative sarcopenia (HR = 2.332, *P* = 0.001), and postoperative sarcopenia (HR = 3.189, *P* = 0.011) were independent risk factors for OS. SML (OS: HR = 11.231, 95%CI: 2.532–31.221, *P* = 0.002; DFS: HR = 10.562, 95% CI: 2.312–40.022, *P* = 0.002) were independent risk factors for five-year DFS and OS in GC patients.	Preoperative sarcopenia is an independent predictor of both short-term and long-term clinical outcomes in GC patients, while significant skeletal muscle loss during curative gastrectomy further worsens OS and DFS.
Lee et al., 2018 (Korea) (36)	*N* = 140 Sarcopenic: 69 Nonsarcopenic: 66	Palliative chemotherapy	**Sarcopenic group:** OS: (6.8 months) **Nonsarcopenic group:** OS: (10.3 months)	31.9 months	Sarcopenia group had a significantly shorter OS than those without (*P* = 0.033).	Sarcopenia can serve as a predictor of poor prognosis in advanced GC patients receiving palliative chemotherapy.
O’Brien et al., 2018 (Ireland) (37)	*N* = 56 68.4 ± 11.9	Surgery	**Sarcopenic group** Serious complications: (55%) **Nonsarcopenic group** Serious complications: (25%)	39.9 months	Sarcopenia was significantly associated with decreased OS (*P* = 0.003) and served as an independent adverse predictor of OS (HR = 10.915; *P* = 0.001) as well as a predictor of serious in-hospital complications (OR = 3.508; *P* = 0.042).	Sarcopenia was significantly associated with decreased OS and serious postoperative complications in patients undergoing radical gastrectomy. We recommend incorporating preoperative CT-based skeletal muscle index assessment into routine clinical practice.
Wu et al., 2025 (China) (38)	*N* = 1654 66 (14)	Surgery	Overall complication (25.6%) Severe complications (5.9%)	60.9 months	Low muscle strength was identified as an predictor for postoperative complications (OR = 1.502, 95% CI: 1.079–2.090, *P* = 0.016), OS (HR = 1.612, 95% CI: 1.224–2.123, *P* = 0.001) and DFS (HR = 1.558, 95% CI: 1.221–1.987, *P* < 0.001).	The combination of low muscle strength with either low muscle mass or low muscle-specific strength demonstrated optimal predictive consistency for postoperative complications and survival outcomes in GC patients.
Wagh et al., 2024 (India) (39)	*N* = 74 55.86	Surgery	**Sarcopenic group** Postoperative complication: (27.59%) **Nonsarcopenic group** Postoperative complication: (25.64%)	30 days	Sarcopenia was not associated with increased risk of major complications (*P* = 0.857).	Sarcopenia, though associated with a substantial proportion of patients with GC, does not significantly affect early postoperative complications in a high volume oncology centre.
Beuran et al., 2018 (Romania) (40)	*N* = 78 67.7	Surgery	Mortality (7.7%)	30 days	Sarcopenia showed significant correlations with both overall complication rates (*P* < 0.05) and surgical site infection rates (*P* < 0.01).	Sarcopenia is highly prevalent in patients having surgery for GC in Romania and correlates with increased postoperative morbidity.
Bhattacharyya et al., 2022 (India) (41)	*N* = 72 55.67 ± 11.15	Surgery	**Sarcopenic group** T3 tumors: (65.7%); T4 tumors: (8.6%) **Nonsarcopenic group** T3 tumors: (41.7%); T4 tumors: (2.8%)	NA	Sarcopenic patients were having higher T3/T4 tumors as compared to non-sarcopenic patients (*P* = 0.04; *P* = 0.001).	Sarcopenia is an independent prognostic factor for adverse short-term postoperative outcomes in GC patients.
Chen et al., 2024 (China) (42)	*N* = 289 67.6 ± 11.4	Surgery	**SO group** 5-year OS: (6.74%); 3-year DFS (6.74%) **NSO group** 5-year OS: (82.84%); 3-year DFS (81.82%)	60 months	SO is an independent predictive factor for both 5-year OS (HR = 13.529, 95%CI: 7.064–25.912, *P* < 0.001) and DFS (HR = 13.387, 95% CI: 6.991–25.634, *P* < 0.001) in GC patients following surgery.	Preoperative assessment of SO is useful not only for monitoring nutritional status but also for predicting 5-year OS in gastrointestinal cancer patients.
Zhao et al., 2025 (China) (43)	*N* = 335 67 (15)	Surgery	**Excessive SMI loss group** Total complications (32.1%) Severe complications (14.3%) Hospital stay (15 days); Costs (75, 326.8¥) **Non-excessive SMI loss group** Total complications (18.6%) Severe complications (6.0%) Hospital stay (16 days); Costs (62, 354.2¥)	30 days	Excessive SMD loss (OR = 1.864; 95% CI: 1.052–3.300; *P* = .033) and excessive SMI loss (OR = 1.853; 95% CI: 1.038–3.306; *P* = .037) were independent risk factors for total postoperative complications.	Both excessive losses in SMI and SMD are independently associated with the incidence of postoperative complications. Clinical interventions targeting modifiable preoperative risk factors are essential to minimize perioperative muscle loss and improve prognosis.
Rodrigues et al., 2021 (Spain) (44)	*N* = 198 73.5	Surgery	**Sarcopenic group** Postoperative complication: (44.4%) **Nonsarcopenic group** Postoperative complication: (39.8%)	54.5 months	No body composition category was found to be associated with postoperative complications or worse OS and DFS (*P* > 0.05).	Neither sarcopenia nor sarcopenic obesity was associated with adverse postoperative outcomes in GC patients.

### 3.2 Prevalence and assessment of sarcopenia in gastric cancer patients

This systematic review synthesizes evidence of a 6.8%–72.22% sarcopenia prevalence in gastric cancer patients, with assessment metrics including SMI (12, 14–24, 26–28, 30–32, 34–44), IMAC (12, 30), VFA (21, 30, 42, 44), VAT (15), PMI (29), BMI (26), physical performance (13, 14, 19, 20, 23, 38, 39, 41), body composition (25), SMD (23, 43), muscle strength (13, 14, 19, 20, 23, 38, 39, 41), and muscle-specific strength (38), where CT emerged as the predominant diagnostic modality implemented alongside criteria from the EWGSOP (13, 14, 19, 20, 23, 25, 38, 43), AWGS (19, 20, 38, 39), KNHANES (38), WHO (26), and international consensus definitions (17), while VAT specifically serves as a biomarker for sarcopenic obesity with thresholds at −150 to −50 HU measured through volumetric analysis (12), dynamometry (13, 14, 19, 20, 38, 39), multifrequency BIA (25), and handheld dynamometer (41), revealing significant heterogeneity in threshold definitions across instruments and inconsistent cutoffs for identical tools (**Table 3**).

### 3.3 Survival and prognosis of gastric cancer

Analysis of gastric cancer outcomes in the included literature primarily focused on postoperative complications, encompassing overall complications (24, 27, 38) and major complications (24, 37); survival metrics including OS (16–18, 21, 22, 24, 26, 28, 29, 32–36, 42), DFS (24, 34, 42), and RFS (18, 21, 22, 34); as well as hospitalization duration and costs (17, 18, 20, 23, 43). Two additional studies evaluated sarcopenia’s impact on chemotherapy delays (43) and treatment-related toxicities (31) in gastric cancer patients. Evidence indicates significantly elevated overall complication rates among sarcopenic patients, with major complication rates reaching 12.9%–43.8% in this subgroup. Regarding survival outcomes, sarcopenia substantially reduced long-term survival rates and increased recurrence risk. Sarcopenic patients incurred higher hospitalization costs with prolonged hospital stays. Follow-up durations varied considerably across studies: short-term (30-day) assessments (12–14, 20, 25, 39, 40, 43), intermediate term (3–5 years) (15–17, 21, 23, 24, 26, 28, 32–38, 42, 44), and long term (18, 22). Regarding short-term outcomes, sarcopenia substantially increases postoperative complication risks, including infectious complications (12, 25, 30), anastomotic leakage (12, 30), and major complications (19, 24); prolongs hospital stays (17, 20, 23); and elevates healthcare costs (20, 23). Sarcopenia independently predicts reduced survival, significantly diminishing 5-year overall survival (16, 18, 42) and disease-free survival (18, 24), with particularly pronounced effects in metastatic/advanced disease (33, 36). Concurrent evidence indicates sarcopenia correlates with higher chemotherapy-related toxicities (31) and treatment delay risks (34). However, two studies reported no significant impact of sarcopenia or body composition alterations on postoperative complications or survival outcomes (39, 44) (**Table 3**).

### 3.4 Insights into the mechanism of action between gastric cancer and sarcopenia

**Figure 2:**
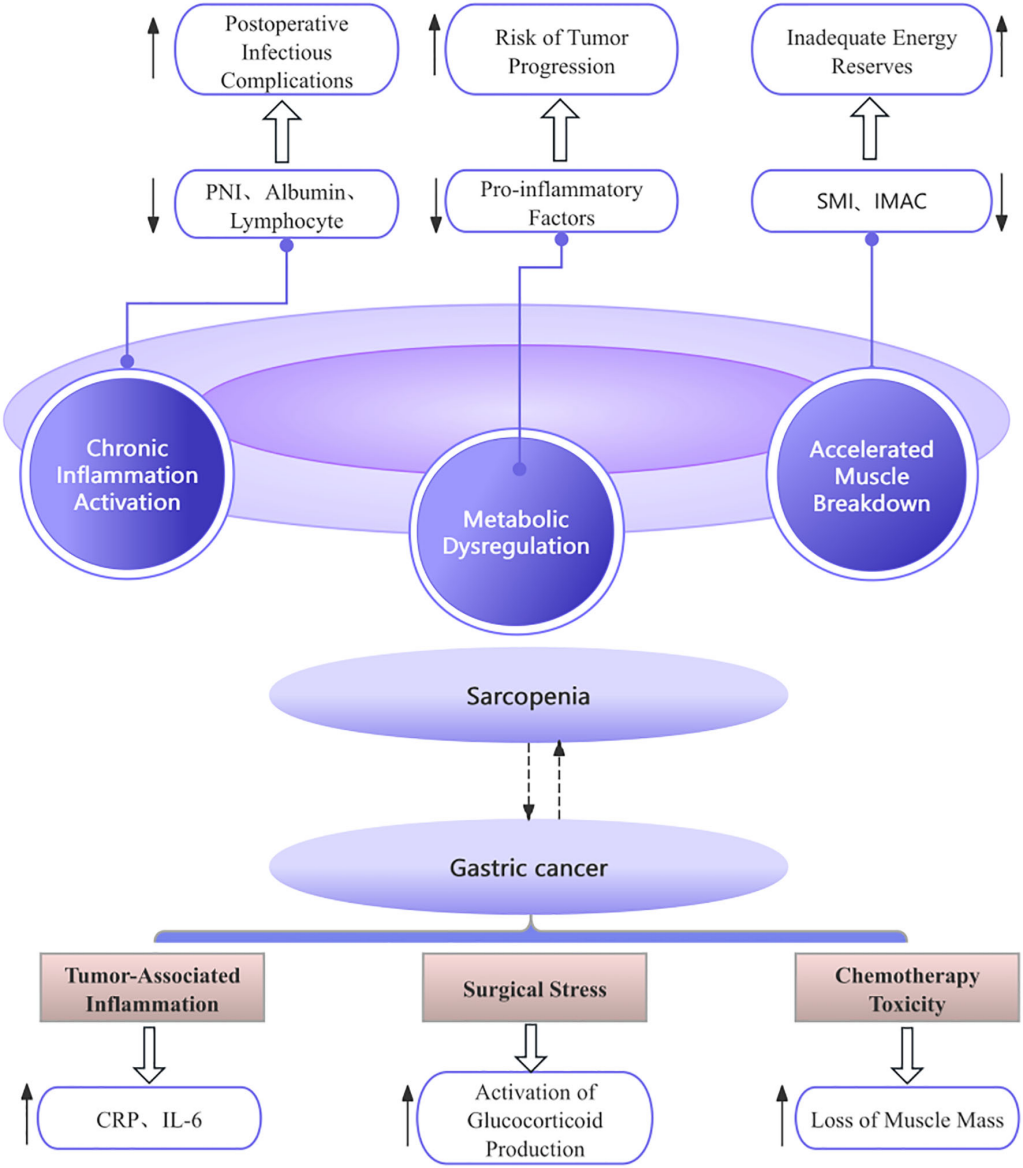
Diagram of the mechanism of action. PNI, Prognostic Nutritional Index; SMI, Skeletal Muscle Index; IMAC, intramuscular adipose tissue content.

Through a review of the included literature, we have preliminarily summarized that the core mechanisms underlying the interaction between sarcopenia and gastric cancer may encompass four key aspects. First, sarcopenia exacerbates postoperative risks in gastric cancer patients through disordered nutritional metabolism (12, 14, 16, 19). Second, inflammation and immune suppression mediate bidirectional adverse effects (12, 16, 18, 20). Third, surgical stress and tumor progression act synergistically to cause harm (13, 17, 18, 21). Finally, the fourth aspect may involve the compounded risk resulting from altered body composition, such as sarcopenic obesity (12, 15, 18, 19). Please refer to the schematic diagram of the mechanism of action for specific details (**Figure 2**).

## 4 Discussion

Gastric cancer treatment risks increase with advancing age, while the prevalence of sarcopenia is notably higher in older populations (45). Therefore, precise nutritional risk assessment and careful selection of management strategies are clinically essential for elderly patients. Multiple studies have confirmed that sarcopenia is a significant risk factor for survival following curative surgery for gastric cancer (46, 47), although the exact relationship between sarcopenia and gastric cancer remains incompletely understood. Consistent with the majority of existing evidence, this review confirms that sarcopenia significantly increases the risk of postoperative complications, reduces overall survival (OS) and disease-free survival (DFS), and is associated with prolonged hospitalization and higher medical costs (37, 43). Notably, one study reported a fivefold increase in the risk of major complications among sarcopenic patients compared to non-sarcopenic patients after adjusting for covariates (19). Li et al. (35) identified both preoperative and postoperative sarcopenia as independent risk factors for OS in gastric cancer patients (35). However, conflicting evidence exists regarding the effect of sarcopenia on OS (48). There is growing research interest in the impact of sarcopenic obesity on gastric cancer outcomes (20, 21). Due to the complexity of screening procedures, clinicians often rely excessively on BMI for nutritional assessment, which may lead to underrecognition of nutritional risks in overweight or obese patients (49).

However, several limitations persist in current research, including the absence of standardized diagnostic criteria for sarcopenia—particularly in the systematic assessment of muscle strength and physical performance—which contributes to substantial discrepancies in reported prevalence rates. Most existing studies depend on preoperative imaging for muscle mass evaluation, with CT being the most widely used modality, despite ongoing debate regarding its validity in accurately reflecting whole-body musculature (25). Bioelectrical impedance analysis (BIA), although radiation-free and cost-effective, has not been routinely incorporated into preoperative assessment protocols (50). Moreover, muscle strength evaluations such as grip dynamometry can be influenced by subjects’ volitional effort or preexisting hand pathologies, potentially affecting measurement accuracy (51). Current sarcopenia diagnosis predominantly relies on SMI cutoffs; however, conventional definitions often overlook age as a critical modifier of muscle quality. Excessive reliance on SMI alone may therefore introduce bias into research outcomes (52). The revised 2018 EWGSOP guidelines explicitly incorporated “reduced muscle quality” as a core diagnostic criterion (53), underscoring the inadequacy of muscle quantity alone in predicting gastric cancer prognosis and highlighting the necessity of incorporating comprehensive functional assessments. A recent multicenter study integrating CT imaging and clinical data from three prospective gastric cancer cohorts proposed a composite metric known as the SMG, which synergistically evaluates both muscle mass and quality and may outperform single-parameter indices (54). This approach is conceptually analogous to diamond valuation, which considers both carat weight and clarity; nevertheless, the utility of SMG remains underexplored in gastric oncology. Concurrently, IMAC has emerged as a quantifiable biomarker of muscle quality, with emerging evidence indicating that IMAC-guided prehabilitation programs may contribute to improved surgical outcomes (12).

The pathophysiological interplay between gastric cancer and sarcopenia involves complex mechanisms, with no definitive causal relationship yet established. Current evidence indicates that gastric cancer patients exhibit heightened susceptibility to sarcopenia, primarily mediated through accelerated protein catabolism, systemic inflammatory responses, metabolic dysregulation, and reduced nutritional intake—processes intrinsically linked to cancer cachexia (55). Cachexia further impairs skeletal muscle regenerative capacity (56), while the concomitant loss of muscle-derived myokines, which exert anti-inflammatory and anti-tumor effects, may facilitate cancer progression (57). The immunometabolic imbalance hypothesis posits that fat-infiltrated skeletal muscle secretes aberrant adipokines that suppress NK cell function, thereby exacerbating postoperative immunosuppression and predisposing patients to infectious complications (58). Notably, males with sarcopenic obesity demonstrate elevated perioperative risk, largely attributable to increased adipose tissue friability that compromises surgical exposure (59). Concurrently, inadequate protein reserves in sarcopenic patients impair postoperative tissue repair under hypercatabolic stress, leading to delayed wound healing and prolonged hospitalization (60, 61). Skeletal muscle serves as an amino acid reservoir that mobilizes substrates for biosynthetic defense during surgical trauma; sarcopenia-induced amino acid deficiency restricts this reparative capacity, thereby increasing infection susceptibility (62). Importantly, most available studies do not adequately evaluate post-gastrectomy dietary intake patterns, which precludes definitive attribution of muscle loss to either surgical sequelae or underlying cancer pathophysiology (29).

This review synthesizes current evidence regarding the association between gastric cancer and sarcopenia, delineating the prevalence of sarcopenia among gastric cancer patients and evaluating its prognostic implications. While consolidating key insights, several limitations must be acknowledged. First, the predominance of retrospective study designs inherently constrains the assessment of core diagnostic parameters for sarcopenia—such as muscle strength and physical performance—which are frequently unavailable in archival datasets. Second, a geographical selection bias is evident, with studies predominantly involving East Asian populations and a notable scarcity of data from Western demographics. Furthermore, the use of heterogeneous assessment metrics across studies complicates comparative analysis. To address these issues, future efforts should focus on establishing integrated diagnostic criteria that combine artificial intelligence—enhanced imaging with validated biomarkers. Such advances would improve diagnostic accuracy and facilitate the identification of patients who may benefit from early nutritional and therapeutic interventions aimed at increasing muscle mass and improving clinical outcomes. Additionally, mechanistic studies are needed to elucidate the role of muscle density in gastric cancer progression, alongside randomized controlled trials to determine whether targeted interventions for sarcopenia significantly improve long-term survival.

## 5 Conclusion

This study demonstrates that sarcopenia is highly prevalent after gastric cancer surgery and serves as a significant predictor of adverse postoperative outcomes. However, its underlying mechanisms and standardized diagnostic criteria require further elucidation. Methodological variations and the lack of uniform assessment metrics across existing studies have contributed to inconsistent conclusions. Future efforts should focus on developing muscle preservation strategies and multimodal diagnostic approaches that integrate both mass and functional parameters to improve diagnostic accuracy and clinical outcomes. Moreover, prospective studies are essential to establish causal relationships between sarcopenia and gastric cancer progression, thereby facilitating evidence-based clinical pathways.

## Data Availability

The original contributions presented in the study are included in the article/supplementary material. Further inquiries can be directed to the corresponding author.
